# Supplementary material for: Chemical Constituents and Antimicrobial Activity of a Ganoderma lucidum (Curtis.) P. Karst. Aqueous Ammonia Extract
Source: Plants (Basel). 2023 Jun 11;12(12):2271. doi: 10.3390/plants12122271 (PMC10305452; doi:10.3390/plants12122271)
Supplement: Supplementary file 1 [file plants-12-02271-s001.zip › plants-2399080-supplementary.pdf]

Chemical Constituents and Antimicrobial Activity of a *Ganoderma lucidum* (Curtis.) P. Karst. Aqueous Ammonia Extract

Eva Sánchez-Hernández, Ana Teixeira, Catarina Pereira, Adriana Cruz, Jesús Martín-Gil, Rui Oliveira and Pablo Martín-Ramos

SUPPORTING INFORMATION

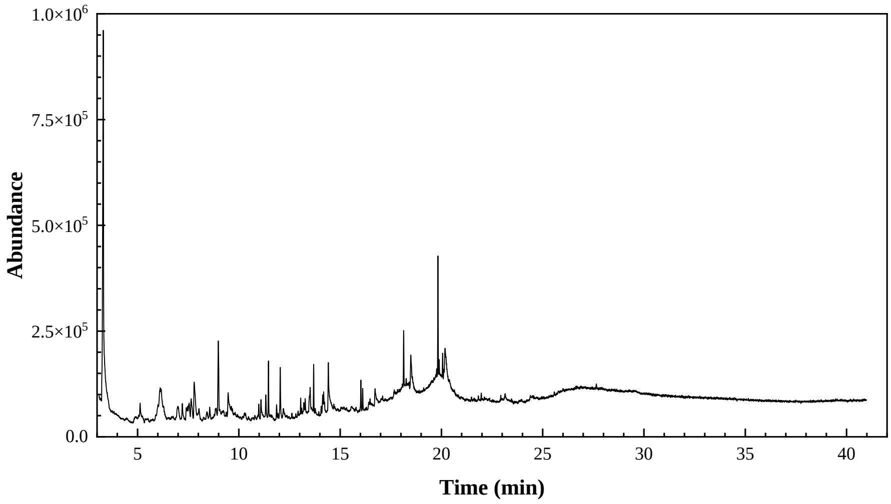

Figure S1. GC-MS chromatogram of *G. lucidum* carpophore aqueous ammonia extract.

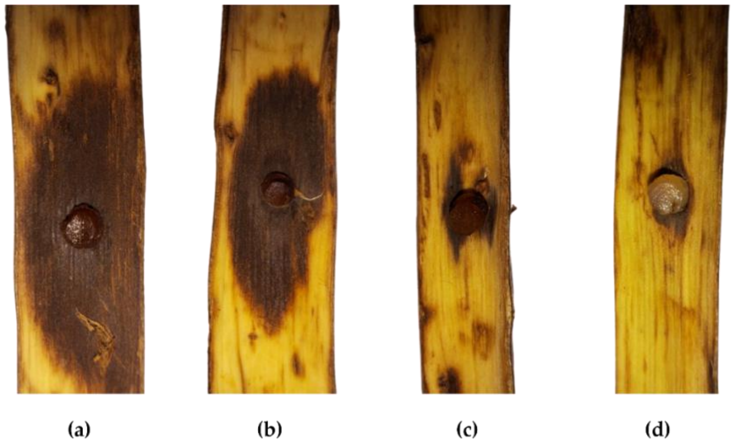

Figure S2. Canker lengths observed in holm oak excised stems artificially inoculated with *P. cinnamomi*: (**a**) control, no treatment; (**b**) treated with the COS–*G. lucidum* carpophore extract conjugate complex at MIC = 78.12 μg·mL^{-1}; (**c**) MIC×5 = 390.6 μg·mL^{-1}; (**d**) MIC×10 = 781.2 μg·mL^{-1}. Only one replicate per treatment is shown.

Table S1. Antimicrobial activity reported in the literature for *G. lucidum* extracts.

Collection site	Solvent extraction	Microorganism	Effectiveness	Ref.
Spain	Ammonia + water	*Botryosphaeria dothidea*	MIC = 500 μg·mL^{-1}	This work
		Diplodia corticola	MIC = 500 μg·mL^{-1}	
		Dothiorella iberica	MIC = 750 μg·mL^{-1}	
		Phytophthora cinnamomi	MIC = 187.5 μg·mL^{-1}	
Serbia	Methanol	*Staphylococcus aureus*	MIC = 150 μg·mL^{-1}	[1]
		Bacillus cereus	MIC = 17 μg·mL^{-1}	
		Micrococcus flavus	MIC = 150 μg·mL^{-1}	
		Listeria monocytogenes	MIC = 150 μg·mL^{-1}	
		Pseudomonas aeruginosa	MIC = 17 μg·mL^{-1}	
		Salmonella typhimurium	MIC = 35 μg·mL^{-1}	
		Escherichia coli	MIC = 300 μg·mL^{-1}	
		Enterobacter cloacae	MIC = 17 μg·mL^{-1}	
		Aspergillus fumigatus	MIC = 70 μg·mL^{-1}	
		Aspergillus versicolor	MIC = 35 μg·mL^{-1}	
		Aspergillus ochraceus	MIC = 150 μg·mL^{-1}	
		Aspergillus niger	MIC = 600 μg·mL^{-1}	
		Trichoderma viride	MIC = 70 μg·mL^{-1}	
		Penicillium funiculosum	MIC = 150 μg·mL^{-1}	
		Penicillium ochrochloron	MIC = 300 μg·mL^{-1}	
		Penicillium aurantiogriseum	MIC = 600 μg·mL^{-1}	
China		*S. aureus*	MIC = 70 μg·mL^{-1}	
		B. cereus	MIC = 35 μg·mL^{-1}	
		M. flavus	MIC = 100 μg·mL^{-1}	
		L. monocytogenes	MIC = 150 μg·mL^{-1}	
		P. aeruginosa	MIC = 70 μg·mL^{-1}	
		S. typhimurium	MIC = 70 μg·mL^{-1}	
		E. coli	MIC = 150 μg·mL^{-1}	
		E. cloacae	MIC = 70 μg·mL^{-1}	
		A. fumigatus	MIC = 1000 μg·mL^{-1}	
		A. versicolor	MIC = 150 μg·mL^{-1}	
		A. ochraceus	MIC = 100 μg·mL^{-1}	
		A. niger	MIC = 1500 μg·mL^{-1}	
		T. viride	MIC = 70 μg·mL^{-1}	
		P. funiculosum	MIC = 70 μg·mL^{-1}	
		P. ochrochloron	MIC = 150 μg·mL^{-1}	
		P. aurantiogriseum	MIC = 300 μg·mL^{-1}	
Poland	Ethanol + water	*Staphylococcus epidermidis*	MIC = 2500 μg·mL^{-1}	[2]
		S. aureus	MIC = 1250−5000 μg·mL^{-1}	
		Bacillus subtilis	MIC = 2500 μg·mL^{-1}	
		Micrococcus luteus	MIC = 630−1250 μg·mL^{-1}	
		E. coli	MIC = 2500 μg·mL^{-1}	
		Klebsiella pneumoniae	MIC = 1250−2500 μg·mL^{-1}	
		P. aeruginosa	MIC = 1250−2500 μg·mL^{-1}	
		Proteus mirabilis	MIC = 2500 μg·mL^{-1}	
India	Methanol or water (200 mg/mL)	*E. coli*	IZ = 27−28 mm	[3]
		P. aeruginosa	IZ = 20−25 mm	
		Salmonella typhi	IZ = 30−31 mm	
		S. aureus	IZ = 29−31 mm	
		Streptococcus mutans	IZ = 25−27 mm	
		Penicillium spp.	IZ = 20−26 mm	
		A. fumigatus	IZ = 17−24 mm	
		A. niger	IZ = 17−27 mm	
		Aspergillus flavus	IZ = 20−23 mm	
		Mucor indicus	IZ = 23−30 mm	

	Chloroform, ethanol, methanol or water	*A. niger*	MIC = 150−400 µg·mL^{-1}	[4]
		Aspergillus terreus	MIC = 200−450 µg·mL^{-1}	
		Aspergillus sydowii	MIC = 200−350 µg·mL^{-1}	
		Fusarium spp.	MIC = 150−400 µg·mL^{-1}	
		Penicillium spp.	MIC = 150−400 µg·mL^{-1}	
	Acetone, methanol, chloroform or water (100 mg/mL)	*B. subtilis*	IZ = 10.5−21 mm	[5]
		Corynebacterium diphtheriae	IZ = 12−23 mm	
		S. aureus	IZ = 11.5−22 mm	
		P. mirabilis	IZ = n.a.−14 mm	
		E. coli	IZ = 10−15.5 mm	
		K. pneumoniae	IZ = n.a.−16.5 mm	
		S. typhi	IZ = n.a.−17.5 mm	
		P. aeruginosa	IZ = n.a.−12 mm	
	Acetone	*B. subtilis*	MIC = 14,000 µg·mL^{-1}	[6]
		S. aureus	MIC = 20,800 µg·mL^{-1}	
		S. typhi	MIC = 19,000 µg·mL^{-1}	
		E. coli	MIC = 8170 µg·mL^{-1}	
		K. pneumoniae	MIC = 4330 µg·mL^{-1}	
		P. aeruginosa	MIC = 21,300 µg·mL^{-1}	
Pakistan	Acetone, methanol or water (20%)	*Fusarium oxysporum* spp.	IR = 40−57 %	[7]
		Alternaria alternata	IR = 33−52 %	
South Corea	Water	*Bacillus anthracis*	MIC = 3500 µg·mL^{-1}	[8]
		B. cereus	MIC = 2000 µg·mL^{-1}	
		B. subtilis	MIC = 3500 µg·mL^{-1}	
		M. luteus	MIC = 750 µg·mL^{-1}	
		S. aureus	MIC = 1500 µg·mL^{-1}	
		E. coli	MIC = 1750 µg·mL^{-1}	
		Klebsiella oxytoca	MIC = 5000 µg·mL^{-1}	
		K. pneumoniae	MIC = 5000 µg·mL^{-1}	
		Proteus vulgaris	MIC = 1250 µg·mL^{-1}	
		Salmonella thompson	MIC = 5000 µg·mL^{-1}	
		S. typhi	MIC = 2500 µg·mL^{-1}	
		S. typhimurium	MIC = 5000 µg·mL^{-1}	
		Serratia marcescens	MIC = 5000 µg·mL^{-1}	
Iran	Chloroform or hexane	*S. aureus*	MIC = 6250 µg·mL^{-1}	[9]
		B. subtilis	MIC = 6250 µg·mL^{-1}	
		E. coli	MIC > 10,000 µg·mL^{-1}	
		P. aeruginosa	MIC > 10,000 µg·mL^{-1}	
		Candida albicans	MIC = 6250 µg·mL^{-1}	

IR: inhibition rate; IZ: inhibition zone; MIC: minimum inhibitory concentration; n.a.: no activity.

References (reference numbers do not match those in the main text)

1. Stojković, D.S.; Barros, L.; Calhelha, R.C.; Glamočlija, J.; Ćirić, A.; Van Griensven, L.J.; Soković, M.; Ferreira, I.C. A detailed comparative study between chemical and bioactive properties of *Ganoderma lucidum* from different origins. *International Journal of Food Sciences and Nutrition* **2014**, *65*, 42-47.
2. Skalicka-Wozniak, K.; Szypowski, J.; Los, R.; Siwulski, M.; Sobieralski, K.; Glowniak, K.; Malm, A. Evaluation of polysaccharides content in fruit bodies and their antimicrobial activity of four *Ganoderma lucidum* (W Curt.: Fr.) P. Karst. strains cultivated on different wood type substrates. *Acta Societatis Botanicorum Poloniae* **2012**, *81*.
3. Sridhar, S.; Sivaprakasam, E.; Balakumar, R.; Kavitha, D. Evaluation of antibacterial and antifungal activity of *Ganoderma lucidum* (Curtis) P. Karst fruit bodies extracts. *World Journal of Science and Technology* **2011**, *1*, 8-11.
4. Radhika, R.; Rajan, S. Antifungal potentials of *Ganoderma lucidum* extracts. *Plant Cell Biotechnology and Molecular Biology* **2021**, *22*, 22-27.
5. Kamble, R.; Venkata, S.; Gupte, A. Antimicrobial activity of *Ganoderma lucidum* mycelia. *J Pure Appl Microbiol* **2011**, *5*, 983-986.
6. Quereshi, S.; Pandey, A.; Sandhu, S. Evaluation of antibacterial activity of different *Ganoderma lucidum* extracts. *J Sci Res* **2010**, *3*, 9-13.
7. Shahid, A.A.; Asif, M.; Shahbaz, M.; Ali, M. Antifungal potential of *Ganoderma lucidum* extract against plant pathogenic fungi of *Calendula officinalis* L. In Proceedings of 5th International Conference on Biological, Chemical and Environmental Sciences (BCES-2016) March; pp. 24-25.
8. Yoon, S.Y.; Eo, S.K.; Kim, Y.S.; Lee, C.K.; Han, S.S. Antimicrobial activity of *Ganoderma lucidum* extract alone and in combination with some antibiotics. *Arch. Pharm. Res.* **1994**, *17*, 438-442, doi:10.1007/BF02979122.
9. Vazirian, M.; Faramarzi, M.A.; Ebrahimi, S.E.S.; Esfahani, H.R.M.; Samadi, N.; Hosseini, S.A.; Asghari, A.; Manayi, A.; Mousazadeh, S.A.; Asef, M.R. Antimicrobial effect of the Lingzhi or Reishi medicinal mushroom, *Ganoderma lucidum* (higher Basidiomycetes) and its main compounds. *International Journal of Medicinal Mushrooms* **2014**, *16*.
